# Supplementary material for: Comparing infrared spectroscopic methods for the characterization of Plasmodium falciparum-infected human erythrocytes
Source: Commun Chem. 2021 Sep 9;4:129. doi: 10.1038/s42004-021-00567-2 (PMC9814045; doi:10.1038/s42004-021-00567-2)
Supplement: Supplementary file 2 — Reporting Summary [file 42004_2021_567_MOESM2_ESM.pdf]

nature research

Corresponding author(s): Dr Agnieszka BANAS
Dr Rajesh Chandramohanadas

Last updated by author(s): Jul 29, 2021

Reporting Summary

Nature Research wishes to improve the reproducibility of the work that we publish. This form provides structure for consistency and transparency in reporting. For further information on Nature Research policies, see our Editorial Policies and the Editorial Policy Checklist.

Statistics

For all statistical analyses, confirm that the following items are present in the figure legend, table legend, main text, or Methods section.

n/a	Confirmed	
☐	☒	The exact sample size (n) for each experimental group/condition, given as a discrete number and unit of measurement
☒	☐	A statement on whether measurements were taken from distinct samples or whether the same sample was measured repeatedly
☒	☐	The statistical test(s) used AND whether they are one- or two-sided<br>*Only common tests should be described solely by name; describe more complex techniques in the Methods section.*
☒	☐	A description of all covariates tested
☒	☐	A description of any assumptions or corrections, such as tests of normality and adjustment for multiple comparisons
☒	☐	A full description of the statistical parameters including central tendency (e.g. means) or other basic estimates (e.g. regression coefficient) AND variation (e.g. standard deviation) or associated estimates of uncertainty (e.g. confidence intervals)
☒	☐	For null hypothesis testing, the test statistic (e.g. F, t, r) with confidence intervals, effect sizes, degrees of freedom and P value noted<br>*Give P values as exact values whenever suitable.*
☒	☐	For Bayesian analysis, information on the choice of priors and Markov chain Monte Carlo settings
☒	☐	For hierarchical and complex designs, identification of the appropriate level for tests and full reporting of outcomes
☒	☐	Estimates of effect sizes (e.g. Cohen's d, Pearson's r), indicating how they were calculated

Our web collection on statistics for biologists contains articles on many of the points above.

Software and code

Policy information about availability of computer code

Data collection	FTIR: Bruker Hyperion 3000 IR microscope with a FPA detector attached to the Vertex 80v spectrometer (Bruker) OPUS software 7.8 (provided with the Bruker instrument)<br>O-PTIR: mIRage microscope (PSC, USA) PTIRStudio 4.0 software supplied with the instrument.<br>AFM-IR: nanoIR3 instrument (Bruker Nano, USA) Analysis Studio 3.16 provided with the instrument was used to collect all experimental data
Data analysis	R Environment with following libraries: hyperSpec, ggplot2, dendextend, plyr<br>Fiji distribution of ImageJ 2.0.0-rc69 [64-bit]

For manuscripts utilizing custom algorithms or software that are central to the research but not yet described in published literature, software must be made available to editors and reviewers. We strongly encourage code deposition in a community repository (e.g. GitHub). See the Nature Research guidelines for submitting code & software for further information.

Data

Policy information about availability of data

All manuscripts must include a data availability statement. This statement should provide the following information, where applicable:

- Accession codes, unique identifiers, or web links for publicly available datasets
- A list of figures that have associated raw data
- A description of any restrictions on data availability

The datasets generated during and/or analyzed during the current study are available from the corresponding author on reasonable request.

Field-specific reporting

Please select the one below that is the best fit for your research. If you are not sure, read the appropriate sections before making your selection.

- [x] Life sciences
- [ ] Behavioural & social sciences
- [ ] Ecological, evolutionary & environmental sciences

For a reference copy of the document with all sections, see nature.com/documents/nr-reporting-summary-flat.pdf

Life sciences study design

All studies must disclose on these points even when the disclosure is negative.

Sample size	This manuscript mainly focused on comparative sub-micron analysis of plasmodium-infected RBCs using individual cells. For our study, 20 iRBCs and 20 control (healthy/un-infected) RBCs were analysed and compared for vibrational spectral characteristics. Conclusions presented in the paper are a cumulative sum of our consistent observations across all cells tested.
Data exclusions	Spectra with low signal-to-noise ratio have been excluded from further analysis
Replication	Plasmodium infected RBCs used in this study came from several cycles of infections which used different donor blood. For our case study about 20 iRBCs and 20 control (healthy, un-infected) RBCs were taken into account. Conclusions presented in the paper are a cumulative sum of our consistent observations across all cells tested.
Randomization	Samples were allocated into experimental group (Plasmodium infected cells - iRBC) based on their appearance under optical microscope and the assessment of experienced Microbiologist.
Blinding	Our analysis was focused deliberately only on infected cells (iRBC) and control (healthy, un-infected) RBCs.

Reporting for specific materials, systems and methods

We require information from authors about some types of materials, experimental systems and methods used in many studies. Here, indicate whether each material, system or method listed is relevant to your study. If you are not sure if a list item applies to your research, read the appropriate section before selecting a response.

Materials & experimental systems

n/a	Involved in the study	
x		Antibodies
	x	Eukaryotic cell lines
x		Palaeontology and archaeology
	x	Animals and other organisms
x		Human research participants
x		Clinical data
x		Dual use research of concern

Methods

n/a	Involved in the study	
x		ChIP-seq
x		Flow cytometry
x		MRI-based neuroimaging

Eukaryotic cell lines

Policy information about cell lines

Cell line source(s)	Human Blood as host cells for plasmodium was purchased as a commercial product from Interstate Blood Bank (USA).
Authentication	None of the cell lines used were authenticated
Mycoplasma contamination	Authors confirm that cells used in the study were devoid of mycoplasma contamination
Commonly misidentified lines (See ICLAC register)	This point is not relevant to our study.

Animals and other organisms

Policy information about studies involving animals; ARRIVE guidelines recommended for reporting animal research

Laboratory animals	This point is not relevant to our study as we analyzed only infected RBCs and control (healthy, un-infected) RBCs.
Wild animals	This point is not relevant to our study as we analyzed only infected RBCs and control (healthy, un-infected) RBCs.

Field-collected samples	Microorganisms were used in this study (3d7 strain of Plasmodium falciparum) made available through MR4.
Ethics oversight	No ethical approval was required as we analyzed only infected RBC bought from Interstate Blood Bank (USA)

Note that full information on the approval of the study protocol must also be provided in the manuscript.
